# Power and type I error rate of false discovery rate approaches in genome-wide association studies

**DOI:** 10.1186/1471-2156-6-S1-S134

**Published:** 2005-12-30

**Authors:** Qiong Yang, Jing Cui, Irmarie Chazaro, L Adrienne Cupples, Serkalem Demissie

**Affiliations:** 1 Department of Biostatistics, Boston University School of Public Health, Boston, MA 02118 USA; 2 Department of Medicine, Boston University School of Medicine, Boston, MA 02118 USA; 3 Department of Mathematics and Statistics, Boston University, Boston, MA 02215 USA

## Abstract

In genome-wide genetic studies with a large number of markers, balancing the type I error rate and power is a challenging issue. Recently proposed false discovery rate (FDR) approaches are promising solutions to this problem. Using the 100 simulated datasets of a genome-wide marker map spaced about 3 cM and phenotypes from the Genetic Analysis Workshop 14, we studied the type I error rate and power of Storey's FDR approach, and compared it to the traditional Bonferroni procedure. We confirmed that Storey's FDR approach had a strong control of FDR. We found that Storey's FDR approach only provided weak control of family-wise error rate (FWER). For these simulated datasets, Storey's FDR approach only had slightly higher power than the Bonferroni procedure. In conclusion, Storey's FDR approach is more powerful than the Bonferroni procedure if strong control of FDR or weak control of FWER is desired. Storey's FDR approach has little power advantage over the Bonferroni procedure if there is low linkage disequilibrium among the markers. Further evaluation of the type I error rate and power of the FDR approaches for higher linkage disequilibrium and for haplotype analyses is warranted.

## Background

Single-nucleotide polymorphisms (SNPs) are the most frequent types of polymorphisms and are commonly used in association mapping of candidate genomic regions. With the completion of the whole human genome sequence and the reduction of costs in SNP genotyping, genome-wide studies with a dense SNP map consisting of 200,000 to 1 million SNP markers will become available in the near future [1]. How to efficiently control the false positive rate, or type I error rate, when a large number of tests are conducted in a genome-wide study is a challenging problem.

For multiple testing problems, the chance to have a false significant test is higher than the nominal level even if each test is controlled at that nominal level. It is therefore of importance to control the probability of having one or more false significant tests. This probability is commonly referred as the family-wise error rate (FWER). That can be written as P(V > 0), where V is the number of rejections of truly null hypotheses. There can be different types of controls for FWER: weak, exact, and strong, corresponding to conditioning on A = all null hypotheses are true (P(V > 0|A)), B = the exact set of truly null hypotheses (P(V > 0|B)), and C = any subset of null hypotheses are true (P(V > 0|C)), respectively. While it is most desirable to have exact control of FWER, it is impossible to calculate a *p*-value because the exact set of truly null hypotheses is unknown. Weak control and strong control are possible to evaluate, but the former may not be sufficient and the latter may be too conservative.

The simplest solution to the multiple testing problems is to apply the Bonferroni procedure, where each test is controlled at *α*/*m*, with *m *being the total number of tests. The Bonferroni procedure yields a strong control of FWER at α, but it could be too conservative when many tests are dependent. Resampling procedures, for example, minP [2], is less conservative than the Bonferroni procedure. The minP procedure generally provides weak control of FWER, and under certain conditions, also renders a strong control of FWER. However it is not always possible to examine the conditions and the resampling could be computationally demanding when there are a large number of tests.

Recently proposed false discovery rate (FDR) procedures by Benjamini and Hochberg [3] and by Storey [4], do not require resampling. Instead of controlling FWER, the FDR procedures control for a less restrictive measure, FDR, i.e., the expected proportion of truly null hypotheses among all the rejected null hypotheses. FDR can be written as E(V/R), where R is the total number of rejected null hypotheses. Both approaches give strong control of FDR and weak control of FWER. A distinction between Storey's and Benjamini's approaches is that the former incorporates an estimated proportion of truly null hypotheses while the latter assumes all hypotheses are truly null. As a result, the FDR approach of Storey is more powerful as a result [4].

There are few published simulation studies that compare FDR procedures with existing approaches for genome-wide association studies. In this work, we will evaluate the power and type I error rate of FDR approach in the context of genome-wide association tests with closely spaced markers using the Genetic Analysis Workshop 14 (GAW14) simulated datasets.

## Methods

### Subjects

We used all 100 simulated replicate datasets of the Danacaa population of GAW14. In each dataset, there were 100 nuclear families, each with an average of 6 members in which at least 2 were affected. There were a total of 1,469 genetic markers used in this study, including 416 microsatellite markers spaced approximately 7 cM apart, 917 SNPs spaced approximately 3 cM apart, and an additional 136 SNPs spaced approximately 0.3 cM apart in the region of disease loci D1, D2, D3, and D4.

In the Danacaa population, only disease loci D1, D2, and D5 (D5 was a marker) were related to the disease status.

### Association analyses

A family-based association test (FBAT) was carried out at each genetic marker using the FBAT program . In FBAT, a score statistic capturing the covariance between the phenotype and marker genotype is computed and standardized by its mean and variance conditional on the parental marker genotype data to adjust for population admixture. The score statistic is distributed as chi-square with degree of freedom equal to the number of different alleles at the marker minus one.

### FDR-adjusted *p*-value: *q*-value

To adjust for multiple testing, for each dataset all the *p*-values from FBAT tests for each dataset were input into the QVALUE program  to compute FDR adjusted *p*-values, called *q*-values. The *q*-value of the j^th ^test with *p*-value *p*_*j *_is defined as , and  is the estimated proportion of truly null hypotheses; *m*is the total number of hypothesis tests. Because the *p*-values of truly null hypotheses are uniformly distributed, one can imagine that most of the large *p*-values, say, *p *> λ, are corresponding to truly nulls. Thus, *π*_0 _can be estimated as , where *λ *is chosen using a smoother method or a bootstrap method to balance the bias and variance of [4]. A null hypothesis will be rejected if the corresponding *q*-value is less than or equal to α.

### Estimation of FDR, FWER, and power

FDR, FWER, and power were estimated using the results of the 100 simulated datasets. To evaluate FWER when all the null hypotheses are true (i.e., *m*_0 _= *m*, where *m*_0 _= *π*_0_·*m *is the number of truly null hypotheses), we used an arbitrary phenotype, that was a random 0–1 variable not associated with any marker. To evaluate the power and FWER when not all null hypotheses are true (i.e., *m*_0 _<*m*), we used disease status as the phenotype in the analyses.

FDR was estimated as , where *N *is the total number of datasets with at least one significant test, i.e., *q*-value ≤ α. FWER was estimated as the proportion of datasets with at least one significant test at SNPs that were not associated with the phenotype. Two measures of power were evaluated. One was the expected number of true positive tests, i.e., E(T), where T = R-V. The other was the probability of at least one true positive test, i.e., P(T > 0). The E(T) was estimated by the average number of detected true associations for each dataset. The P(T > 0) was estimated by proportion of 100 datasets in which at least one true associations was detected. Because the relationship between SNPs in a disease locus region and the disease locus was established through adding disease allele to a subset of haplotypes of the SNPs in generating the data, the association between individual SNPs and disease locus was not completely transparent. Because there was no linkage disequilibrium (LD) in the D1 region, we only count associations with any of the 12 SNPs whose haplotypes were used in generating disease-carrying haplotypes in D2 region, or with D5 itself (which was a marker) as true associations. There may be a few of the 12 SNPs not associated with disease but included in the counting of true association. Because the number of such SNPs is very small compared to the total number of tests (*n *= 1,469), it should have minimal effect on our estimates.

## Results

### FDR of Storey's FDR approach

The estimated FDRs for *m*_0 _= *m *and for *m*_0 _<*m *are presented in Table 1 for Storey's FDR approach. The estimated FDR under both conditions was close but slightly higher than α levels.

### FWER of Bonferroni and Storey's FDR approaches

The estimated FWERs for the Bonferroni and Storey's FDR approaches are presented in Table 2. When *m*_0 _= *m*, the estimated FWER of the two methods were identical and equally conservative. When *m*_0 _<*m*, the FWER of the Bonferroni method was slightly inflated, but the FWER of Storey's FDR approach was much inflated.

### Power of Bonferroni and Storey's FDR approaches

The power of the Bonferroni and Storey's FDR approaches is presented in Table 3. Storey's FDR approach showed slightly better power than the Bonferroni approach.

## Discussion

In this study, we have evaluated the FDR, FWER, and power of Storey's FDR approach, for a genome-wide association study with a closely spaced marker map. We found FDR was slightly inflated for Storey's FDR approach based on 1,469 tests that are mostly independent using 100 simulated datasets. We noted that Storey's FDR approach only yielded weak control of FWER while the Bonferroni method provided close to strong control of FWER. For these simulated dataset, the power of Bonferroni and Storey's FDR approaches was comparable, although the latter yielded slightly higher power at the price of more false positives at markers that were not associated with the disease status.

Most markers in this study were spaced at approximately 3 cM apart and thus there was low LD among them. That may be one of the reasons that the power of Storey's FDR approach is not much higher than the Bonferroni method as expected. Another possible reason is that Storey's FDR approach was originally proposed in the context of microarray data analyses where many null hypotheses were not true. For current study, only three loci contributed to the disease, and only a few surrounding SNPs may be associated with the disease loci.

Unlike single testing problem in which type I error is uniquely defined as the probability that the *p*-value ≤ α given that the null hypothesis is true, there is no unique definition for type I error rate for multiple testing problem. The FWER may be the most commonly used type I error measure for multiple testing problems. But even for FWER, there can be different types of control. The FDR is a valuable addition to the measures for type I error rate for multiple testing problems. Understanding which measure a multiple testing procedure can control is fundamental for appropriately using the procedures and interpreting the results.

For dependent tests, Storey's FDR approach is only valid under weak dependence and with a large number of tests. Weak dependence can be described as a form of dependence that almost does not affect the validity of the approach as the number of tests increases to infinity. An example of weak dependence is dependence in finite blocks. As a rule of thumb, several thousand tests are needed to obtain reasonable estimate of *q*-values and thus control of FDR [5].

For tests that do not satisfy weak dependence, the properties of FDR approaches may be unclear. In this case, resampling methods such as the improved step-down minP algorithm by Ge et al. [6] may be more promising to balance type I error and power. One such scenario is haplotype analyses in genome-wide genetic studies, where SNPs are analyzed by group according to a moving window or LD blocks. Because the groups may have some SNPs in common, many of the tests can be dependent and the dependence among the tests can be very complicated.

## Conclusion

In conclusion, Storey's FDR approach is more powerful than the Bonferroni procedure if strong control of FDR or weak control of FWER is desired. Storey's FDR approach has little power advantage over the Bonferroni procedure if there is low LD among the markers. Further evaluation of the type I error rate and power of the FDR approaches for higher LD and for haplotype analyses is warranted.

## Abbreviations

FBAT: Family-based association test

FDR: False discovery rate

FWER: Family-wise error rate

GAW14: Genetic Analysis Workshop 14

LD: Linkage disequilibrium

SNP: Single-nucleotide polymorphism

## Authors' contributions

All authors participated in the conception and design of the study, interpretation of the data, and revising the article. QY drafted the article.

## Figures and Tables

**Table 1:** Estimated FDR for Storey's FDR approach. Estimated FDR when none of the SNPs associated with the phenotype (*m*_0 _= *m*) and when some of the SNPs associated with the phenotype (*m*_0 _<*m*) based on the 100 simulated datasets.

α	FDR^a^	
	
	*m*_0 _= *m*	*m*_0 _<*m*
0.01	0.01	0.01
0.05	0.08	0.09
0.1	0.15	0.15
0.15	0.20	0.19

^a^*m *= total number of tests; *m*_0 _= number of truly null hypotheses

**Table 2:** Estimated FWER of Bonferroni and Storey's FDR approaches. Estimated FWER when none of the SNPs associated with the phenotype (*m*_0 _= *m*) and when some of the SNPs associated with the phenotype (*m*_0 _<*m*) based on the 100 simulated datasets.

α	FWER (*m*_0 _= *m*)^a^		FWER (*m*_0 _<*m*)	
		
	Bonferroni	Storey's FDR	Bonferroni	Storey's FDR
0.01	0	0	0.03	0.03
0.05	0.01	0.01	0.08	0.21
0.10	0.03	0.03	0.15	0.37
0.15	0.06	0.06	0.21	0.46

^a^*m *= total number of tests; *m*_0 _= number of truly null hypotheses

**Table 3:** Power of Bonferroni and Storey's FDR approaches. Two measures of power, E(T) and P(T > 0), were estimated. The E(T) was the expected number of detected true associations and P(T > 0) was the probability that at least one true associations was detected.

α	E(T)		P(T > 0)	
		
	Bonferroni	Storey's FDR	Bonferroni	Storey's FDR
0.01	1.0	1.0	0.76	0.78
0.05	1.5	1.7	0.90	0.92
0.10	1.6	2.0	0.93	0.93
0.15	1.7	2.1	0.93	0.95
